# Sociocultural adjustment and social support as predictors for international medical sciences students’ anxiety during COVID-19

**DOI:** 10.1186/s12909-024-05379-1

**Published:** 2024-04-10

**Authors:** Jevgenij Razgulin, Gita Argustaitė-Zailskienė, Raimonda Petrolienė, Kastytis Šmigelskas

**Affiliations:** Department of Health Psychology, Faculty of Public Health, Medical Academy, Lithuanian University of Health Sciences, Tilžės g. 18, LT47181, Kaunas, Lithuania

**Keywords:** International students, Anxiety, Sociocultural adjustment, Social support, COVID-19

## Abstract

**Background:**

The prevalence of anxiety is high among international medical sciences students and it increased even more during the COVID-19 pandemic due to different restrictions and social isolation. Successful sociocultural adjustment and social support could be important factors in overcoming those challenges, however, there is a lack of studies which would investigate the role of those factors among inter- national medical students. This study aimed to assess the role of sociocultural adjustment and social support as predictors for international medical students’ anxiety during COVID-19.

**Methods:**

Two measurements were conducted via self-reported questionnaires which consisted of three scales - SCAS, MSPSS and GAD-7. In total, 82 international medical students participated in both measurements in this longitudinal study.

**Results:**

The findings indicated that 37% of international students had symptoms of moderate or severe anxiety during their first year of studies at university. In the second year, during the COVID-19 pandemic and an official lockdown, 35% of international students had symptoms of moderate or severe anxiety. In addition, this study showed that gender and sociocultural adjustment did not play a role as predictors of students’ anxiety during the second year of studies. However, this study revealed that social support provided by family during the first year of studies, as well as having friends or family members who had been ill with COVID-19 predicted higher levels of anxiety at second measurement, while sociocultural adjustment was an even stronger predictor of anxiety in the second year of studies of international medical students.

**Conclusions:**

This knowledge can help to better understand how international medical students felt during the COVID-19 pandemic and what role the above- mentioned factors played in the students’ anxiety. As the anxiety level is quite high among international medical students, universities and mental health service providers should take it into consideration and help them to overcome those challenges.

## Background

### COVID-19 pandemic and anxiety

The World Health Organization announced a pandemic due to the spread of the COVID-19 virus worldwide on 11 March 2020 [1]. Studies reveal that international students were among the primary groups who were the most impacted by the pandemic of COVID-19 [2]. Notably, during the pandemic, anxiety became a common mental health condition [3] with a high prevalence in the general public [4] among adults [5] and international students in particular [6]. International students were specifically impacted by COVID-19, as they encountered new, unforeseen problems and uncertainties as a result of closed universities, online learning, travel limitations, and other restrictions, as well as worry for their loved ones and their own health.

Moreover, medical students are the population considered to have higher levels of anxiety in general due to the study stress, higher demands and competing environment compared to the students from the other study programs [7, 8]. Additionally, being not only medical students, but also international students leads to more pressure on those students, as they need to deal with different challenges of cross-cultural adjustment and at the same time lacking social support therefore they are classified as a group at risk for the variety of different mental health problems including anxiety [5]. And COVID-19 pandemic could even worsen this situation. Feeling anxious is a natural response to stress, and in certain situations, moderate levels of anxiety can actually be advantageous. Anxiety is related to fear and manifests as a future-oriented state of mood that consists of a complex physiological, affective, cognitive, and behavioral response system associated with preparation for upcoming events or circumstances perceived as threatening [9]. Anxiety serves as a signal of potential hazards, prompting people to be vigilant and ready [10]. However, problems arise when the intensity of stress and anxiety becomes excessively high, leading to an interference with an individual’s daily life, routines, engagements, and overall performance. In a study environment, increased anxiety can reduce the ability to absorb information, its processing and reproducing when needed. Moreover, it might have a negative effect on working memory, it can also interfere with attention, thus leading to a decline in academic achievements [11].

Other important factors that could be affected by COVID-19 and might con- tribute to the anxiety experienced by international medical students are social support and sociocultural adjustment. An analysis of 711 students from 41 countries found that anxiety, social isolation, loneliness, and a lack of in-person interactions with peers and professors were among the top challenges during the COVID-19 epidemic [12].

### Social support

Some international students were cut off from their classmates and even their relatives and friends during the COVID-19 pandemic because of online coursework, travel limitations, and the inability to leave homes and communicate with wider social networks. This social isolation,loss of social interactions and sometimes even social support might be thought to have had a negative impact on their mental health and especially on their anxiety level. It is known that social isolation is a risk factor for international students’ stress level, anxiety and mental health in general. In contrast to social isolation, well-developed social networks and social support are very important factors for individuals to cope with distress. They can also lower the risk of mental health problems, such as anxiety [13, 14]. A study by Li et al. [15] indicated that social support is a major protective factor for mental health in challenging environments and it serves as a buffer against the negative effects of stressors. A recent study [16] conducted in China revealed that strong social support was associated with lower anxiety during the COVID-19 pandemic. Li et al. [15] state that by demonstrating empathy and offering support, friends and family can assist young adults in managing stress and maintaining excellent mental health. Additionally, social support is inversely correlated with the intensity of mental health symptoms and aids in maintaining a sense of control when coping with stressful events and anxiety [17]. International students who study abroad and do not have direct connections with their families and friends back home might receive less social support and therefore might be at higher risk of mental health problems compared with local students [14]. A study conducted in China revealed similar results, that students who had low levels of social support had higher levels of anxiety compared with the students, who had higher social support during the COVID-19 pandemic [18].

### Sociocultural adjustment

Sociocultural adjustment might be another important protective factor for mental health and particularly the anxiety of international medical students who are studying in Lithuania. On March 16 2020, the COVID-19 pandemic and lockdown were announced in Lithuania. Due to the lockdown, studies were organized online [3] and it was a major challenge for international students who were residing in Lithuania at that moment. Some international students were isolated from their new environment far away from their close ones during this difficult period, having a challenging time returning to their native countries. These circumstances might have interfered with their sociocultural adjustment, psychological well-being, and anxiety [19]. Adjusting to a new culture and acculturating is one of the main tasks that any new international student deals with when coming to study in a new sociocultural environment [2]. According to Ward and Kennedy [20], sociocultural adjustment is the ability to adapt well, gain skills needed for the new cultural environment, and manage the interactive features of the host culture. It might be measured by evaluating different problems that immigrants face in their daily lives. Sociocultural adjustment may be understood through learning social skills and through cultural learning [21]. It is a dynamic and changing process, which leads to better adaptation in a new cultural environment [22]. However, if international students lack those skills or this process is hampered by uncertain conditions, including restrictions and social isolation brought on by the pandemic, they might face different problems and more challenges in the new environment which might lead to higher levels of stress and anxiety.

### Current study

To provide the reader with some context the lockdown in Lithuania was announced on 16th March 2020 and studies were stopped for two weeks all over the country. After that, most of the studies were moved to online mode till the end of the study year. After that decision, the majority of international students left for their home countries, and only a minority of students stayed in Lithuania until the end of the academic year. From September 1st, the beginning of the new academic year, studies at the University were organized partly online, and partly on-site. Most of the lectures were given online, whereas practical seminars and tutorials were organized on-site as medical studies are based on a lot of hands-on studies at the University and different clinics.

In general, the students are enrolled to the University after they pass the entrance examination. Studies are organized in English, but they have to learn the Lithuanian language to be able to understand and communicate with the patients. Studies are also based on a Problem-based learning approach and there are many hands-on and simulation training involved in curriculum of the studies. The baseline measurements of the current study happened just before the outbreak of the COVID-19 pandemic in Lithuania, while the second measurement was one year later, after some adjustment to pandemic challenges. Anxiety fluctuates, so it is especially relevant to assess it longitudinally, because the observed anxiety may be caused by social, personal, or pandemic variables.

Therefore, this study aimed to assess the importance of social support and sociocultural adaptation as potentially protective factors against international medical sciences students’ anxiety during COVID-19.When investigating the role of social support as a potential independent predictor of anxiety, we specifically focus on the social support perceived by medical sciences students during their first year, as it may influence their anxiety levels in the second year, thus allowing sufficient time for its potential impact on anxiety to manifest. Additionally, we aim to determine whether the adjustment experienced during the first year, which occurred before the official COVID-19 lockdown, has greater significance for students’ anxiety during their second year of studies compared to the sociocultural re-adaptation during the second year. This re-adaptation occurred as students returned partly to on-site studies in their host countries during the COVID-19 pandemic. Understanding the fluctuations of anxiety levels throughout the academic years, including periods of heightened stress such as a pandemic, along with identifying the risk and protective factors, can empower medical educators to recognize the challenges students encounter and offer essential support when needed. This comprehension enables educators to be more responsive to students' needs and to implement effective strategies for promoting their well-being throughout their educational journey. Most of the previous studies done with international students are cross-sectional, mostly conducted during the first year of studies when the adjustment process is not over yet. Therefore, there is a lack of longitudinal studies that measure how international students’ sociocultural adjustment changes over time. This research is based on longitudinal measurement, so it enables analysis of how sociocultural adjustment and anxiety evolve over the first two years of studies. It gives a stronger basis for causal relationships between those variables. Moreover, this study can reveal how such challenges as COVID-19 and social isolation can impact the adaptation of international medical students.

## Methods

### Participants and procedure

The study was conducted in two waves. The first measurement took place in February–March 2020, and the second in October 2020. In total, 133 international full-time undergraduate students from various countries participated in this study during the first measurement. The first measurement was carried out during international students’ first year of studies, from February to March 2020 in Health Sciences University in Kaunas, Lithuania, just before the official lockdown in Lithuania was announced. The second measurement took place in October 2020. In total, 165 students participated in this measurement during their second year. Out of them, 82 international students aged 22.48 ±3,22 years on average participated in both assessments. The sample size was calculated using the following formula [23]:

$$\textrm{Sample}\ \textrm{size}=\frac{\frac{z^2\ast p\ \left(1-p\right)}{e^2}}{1+\left(\frac{z^2\ast p\ \left(1-p\right)}{e^2N}\right)}$$

Where:

z (the z score)=1.96

e (the margin of error)=10 N (the population size)=301

p (the population proportion)=0.3

The calculation of the sample size revealed that 64 or more measurements are needed to have a confidence level of 95% that the real value is within ±10% of the measured value.

Both times students filled out paper questionnaires in lecture rooms. We measured sociocultural adjustment and anxiety level in the first and second year of studies and perceived social support in the first year of studies.

Participants were studying medical sciences (Medicine, Veterinary Medicine, Odontology). International students came from China, Cyprus, Finland, Germany, India, Ireland, Israel, Norway, Slovakia, Spain, Sweden, Switzerland, Syria, and the UK. Participants also belonged to different religious groups, being mostly Jewish, Christian, and Muslim, or indicating themselves as non-religious. The distribution of the sample by socio-demographic groups is shown in Table 1.

**Table 1:** Main characteristics of the study sample (*n* = 82)

**Demographics**			**Mean**	**SD**
**Age** (at 1st measurement)			22.48	3,22
**Gender**	**Number**	**Percent**	**By gender Male (number)**	**Female (number)**
Male	29	35.4		
Female	53	64.6		
**Study programme**				
Medicine	61	64.6	25	36
Veterinary	16	19.5	2	14
Medicine				
Odontology	5	6.1	2	3
**Region**				
Europe	40	48.8	13	27
Israel	37	45.1	15	22
Asia	5	6.1	1	4
**Religion**				
Jews	28	34.1	12	16
Non-religious	22	26.8	7	15
Christians	18	22	8	10
Muslims	7	8.5	1	6
Hindu	1	1.2	0	1
Other	1	1.2	0	1
Not indicated	5	6.1	1	4

### Instrument

A self-reported questionnaire was used to collect data. Some basic sociodemographic items such as age, gender, country of origin, religion and study programme were included. A question of whether the students or their relatives or friends had been infected with COVID-19 was also added.

The questionnaire consisted of three scales.

The Sociocultural Adaptation Scale (SCAS) was used to assess the sociocul- tural adjustment of the students [24]. As is mentioned in systematic reviews of cross-cultural adjustment this scale is one of the most popular tools used in different countries to measure the sociocultural adjustment of international students [25, 26]

The full scale has 40 items. It can be modified according to sample characteristics; therefore, we only used the first 10 items as has been done previously in many cross-sectional studies and comparative analyses. Each statement is rated on a 5-point scale, ranging from ‘no difficulty’ to ‘extreme difficulty.’ A higher score indicates greater challenges in sociocultural adjustment. The respondents must indicate the amount of difficulty they are facing in everyday life in new sociocultural environment areas such as using the transport system and going shopping, also communication and management of other interactions (e.g., dealing with people of higher status), or getting used to a new pace of life. Examples of such statements could be “Talking about yourself to others”, “Making friends”; and “Understanding jokes and humor”. However, those difficulties are not related to discomfort, embarrassment, and anxiety. In our study, the 10-item scale’s internal consistency in two measurements was 0.81 and 0.79.

The Multidimensional Scale of Perceived Social Support (MSPSS) was used to measure social support [27]. It consists of 12 items. Participants rated the statements using a 7-point Likert-type scale, ranging from “very strongly disagree” to “very strongly agree.” Lower scores on the scale indicate a lower level of perceived social support. Individuals scoring between 1 and 2.9 may be classified as perceiving low received social support; a score falling within the range of 3 to 5 indicates a moderate level of social support, while a score between 5.1 and 7 indicates a high level of social support. The items are grouped into three subscales – family, friends, and significant others. Each subscale consists of an equal number of questions, for example, “I can count on my friends when things go wrong” or “There is a special person who is around when I am in need”. In this study, the internal consistency of the scale in two measurements was 0.92 and 0.93.

The General Anxiety Disorder-7 scale (GAD-7) was used to measure the risk of a general anxiety disorder [28]. The GAD-7 is the most widely used assessment scale for anxiety in clinical practice and research due to its diagnostic reliability and efficiency [29]. It is a self-report scale which consists of 7 items. A higher score shows a higher level of anxiety. A score of 10 or above indicates a cut-off for identifying cases of GAD. Cut-off points of 5, 10, and 15 might represent mild, moderate, and severe levels of anxiety. Examples of items are “Not being able to stop or control worrying”, “Being so restless that it is hard to sit still”, or “Becoming easily annoyed or irritable”. In our study, the scale’s internal consistency during the two measurements was 0.88 and 0.89.

## Data analysis

IBM SPSS Statistics for Windows, Version 27 (IBM Corp., Armonk, N.Y., USA) was used to perform statistical analysis for this research. Arithmetic mean ± (SD) and proportions (%) were presented in this study. Skewness and kurtosis coefficients were used to test the normality of the distribution of data. To measure the internal consistency of SCAS, MSPSS, GAD-7 the Cronbach’s alpha coefficient was used. All instruments had Cronbach’s alpha coefficients above 0.7 which was acceptable for proper reliability.

For inferential analysis, the chi-squared test was used to examine the differences between the genders and the Spearman’s rank correlation coefficient was used to measure the direction and strength of relationships between the ordinal variables, such as anxiety, social support, and sociocultural adjustment. The comparison of two groups was performed using the Student’s t-test for independent samples. The univariate and multivariate linear regression was carried out to analyze the significance of perceived social support and sociocultural adjustment on the anxiety level of second-year international students. The strength of putative predictors was expressed in B (absolute effect size) and Beta (relative effect size).

The statistical significance level was set at *p<*0.05.

## Results

### Descriptives

Descriptive statistics of the main variables are shown in Table 2. In the first measurement, 18.8% of the sample reported having symptoms of moderate anxiety, while 18.8% had symptoms of severe anxiety. In the second measurement, 20,7% of the sample reported symptoms of moderate anxiety and 14,5% had symptoms of severe anxiety.

**Table 2:** Distributions of the main indicators under study

Measure-ment	Indicator (scale)	Mean	SD	Median	Scale range
Year 1	Social support (MSPSS)	5.83	1.10	6.08	1–7
	Family Social Support	6.03	1.30	6.50	1–7
	Friends’ Social Support	5.71	1.30	6.00	1–7
	Special Person Social	5.76	1.35	6.00	1–7
	Support				
	Socio-cultural adjustment	2.03	0.60	1.96	1–5
	(SCAS)				
	Anxiety (GAD-7)	9.06	5.77	8.00	0–21
Year 2	Socio-cultural adjustment	1.95	0.51	1.83	1–5
	(SCAS)				
	Anxiety (GAD-7)	7.84	5.10	6.5	0–21

Comparing by gender, at the time of the first measurement, women reported more symptoms of anxiety than men (*p<*0.05, see Table 3), however, by the second measurement there was no statistically significant difference between genders. Men and women did not differ in their perceived social support and sociocultural adjustment (*p>*0.05).

**Table 3:** Sociocultural adjustment, social support, and anxiety by gender

Variable	Gender Difference		
	Male	Female	
Anxiety, year 1	7.00 *±* 5.3	10.19 *±* 5.7	*t* = −2.47, *p* = 0.016*
Anxiety, year 2	6.86 *±* 4.8	8.38 *±* 5.2	*t* = 1.29, *p* = 0.200
Sociocultural Adjustment, year 1	1.86 *±* 0.6	2.12 *±* 0.6	*t* = −1.94, *p* = 0.056
Sociocultural Adjustment, year 2	1.84 *±* 0.5	2.01 *±* 0.5	*t* = −1.47, *p* = 0.146
Social Support, year 1	5.81 *±* 0.9	5.85 *±* 1.2	*t* = −0.16, *p* = 0.871
Social Support, year 2	5.91 *±* 1.0	6.09 *±* 1.2	*t* = −0.70, *p* = 0.483

### Associations of perceived social support and sociocultural adjustment with anxiety

Based on correlations, it was revealed that students with higher levels of anxiety in the second year of studies reported lower social support from family (*r*=-0.28, *p*=0.010) perceived during the first year of studies as well as a poorer sociocultural adjustment at first (*r*=0.29, *p*=0.010) and second year of their studies (*r*=0.41, *p<*0.001).

### Prediction of anxiety

Based on the bivariate correlations, linear regression was carried out to predict the role of social support, sociocultural adjustment, gender, and having friends or family members who had been ill with COVID-19 on the levels of anxiety at the time of the second measurement (Table 4).

**Table 4:** The role of sociocultural adjustment and social support on anxiety: linear regression analysis

	Model 1				Model 2				Model 3			
	B	Beta	t	p	B	Beta	t	p	B	Beta	t	p
Gender, year 1 and year 2	1.23	0.12	1.05	0.296	1.16	0.11	1.00	0.322	1.07	0.10	0.95	0.346
Social support from family, year 1	−0.77	− 0.20	− 1.53	0.13	− 0.81	− 0.21	− 1.60	0.114	−1.00	− 0.26	−2.03	0.047*
Social support from friends, year 1,	0.02	0.01	0.04	0.969	0.15	0.04	0.26	0.797	−0.02	− 0.01	− 0.03	0.974
Social support from special persons, year 1	−0.29	− 0.08	− 0.48	0.632	− 0.37	− 0.10	− 0.61	0.546	0.18	0.05	0.29	0.772
Sociocultural adjustment, year 1	1.55	0.18	1.61	0.113	1.62	0.19	1.68	0.098	−0.20	−0.02	− 0.17	0.863
COVID-19 in family or friends, year 2					−2.15	−0.21	−1.19	0.059	−2.28	−0.22	− 2.10	0.039*
Sociocultural adjustment, year 2									3.64	0.36	2.54	0.013*

*p < 0.05

Model 1 included perceived social support in the first year and sociocultural adjustment at the time of the first measurement as well as gender. None of these independent variables were statistically significant predictors of anxiety (*p>*0.05). Having friends or family members who had been ill with COVID-19 was added to the Model 2 and there were still no significant predictors (*p>*0.05), even though the trend was that those students whose family or friends had been ill with COVID-19 were more likely to experience higher levels of anxiety (Beta=-0.21, *p*=0.059).

Finally, sociocultural adjustment at the time of the second measurement was added to make the Model 3 for a complex statistical prediction of anxiety. Results indicate that having friends or family members who had been ill with COVID-19 significantly predicted higher anxiety levels at the time of the second measurement (Beta=-0.22, *p*=0.039). A similar effect was found for social support from family at baseline (Beta=- 0.26, *p*=0.047), while the greatest was for sociocultural adjustment at the time of the second measurement – it was the strongest independent predictor of the second-year students’ anxiety (Beta=0.36, *p*=0.013).

## Discussion

This study aimed to assess the role of sociocultural adjustment and social support as predictors for international students’ anxiety during COVID-19. The results of this study reveal that 37% of the international students reported having symptoms of moderate or severe anxiety during their first year of studies, while 35% reported it during their second year of studies. The total prevalence of international students’ anxiety slightly decreased during the second study year, however, the difference was not significant as could be expected. This small decrease in anxiety might be due to the continuing effects of the pandemic, an increased number of COVID-19 cases in total population, and to lockdown.

The level of anxiety among international students in Lithuania was quite high. However, it seemed to be similar to other authors’ study results across different countries. An analysis of other studies conducted before the COVID-19 pandemic revealed that the prevalence of anxiety among international students was between 21% in Kazakhstan [30], 29% in the USA [16], and 49% in Germany [31].

Other studies conducted during the COVID-19 pandemic also showed a high prevalence of anxiety among international students. One study conducted in 9 countries (Poland, Slovenia, Czechia, Ukraine, Russia, Germany, Turkey, Israel and Colombia) revealed that on average 30% of the international students had moderate or severe anxiety symptoms with the lowest percentage in Germany (5%) and Czechia (13%) and the highest prevalence in Turkey (51%) [32]. Anxiety was found in 65% of international students in China during the COVID-19 pandemic and a study by Cao et al. [18] on the role of COVID-19 on Chinese students uncovered that 26% of the students were suffering from anxiety because of COVID-19.

Gender-wise, first-year female students reported more symptoms of anxiety than males, however, by the second year of studies this difference had disappeared. Those findings contradict the results of other studies which showed that females were more mentally vulnerable than males during the COVID-19 outbreak and had a higher prevalence of anxiety [33–36]. However, a retrospective survey by Hendriksen et al. [37] similar to our study revealed that the difference in gender was observed only during the first lockdown at the beginning of the pandemic (from March to May 2020), and it ceased to be significant later in the period of second lockdown from November 2020 to May 2020. Men and women did not differ in their sociocultural adjustment or perceived social support during the first and the second measurement. Future research is warranted on the long-term dynamic of anxiety across genders in periods of crisis.

This study revealed that such independent variable as gender did not play a role as a predictor of anxiety in the second year of studies. In contrast, this study reveals that social support is an important factor and those who perceive more social support from their families during the first year of studies, are more likely to experience lower levels of anxiety. A possible explanation for this might be that perceived support from family members is a protective factor against mental health issues of young adults as their close ones provide needed assistance support and empathy during difficult times such as the COVID-19 pandemic [15]. In contrast, a study by Kolozsvari [38] revealed that even though social support perceived from the family members lowered the level of stress of the local students in Hungary, it was not significant for international students. Unlike the social support perceived from friends, that seemed to gain more importance for the international students in Hungary. In our study, perceived support from friends did not play a role in anxiety of international students.

Moreover, those international students whose family members or friends had been infected with COVID-19 during the second year were more likely to experience higher levels of anxiety. The study by Moscaritolo et al. [2] similarly showed that having relatives or friends infected with COVID-19 was a risk factor for increasing anxiety. However, a study by Fruehwirth et al. [6] revealed that COVID-19 diagnosis and hospitalization of oneself, family members or friends were not associated with increased anxiety symptoms.

Furthermore, the level of sociocultural adjustment in the middle of the first study year did not play a significant role in later anxiety during the pandemic. This result might indicate that such critical, unclear, and frightening circumstances as the COVID-19 pandemic, sudden lockdown, social isolation, and other restrictions might interfere with the normal pace of students’ life and socialization and, as a result, the typical process of sociocultural adjustment is disrupted. Later, when the studies return to onsite mode, students have to readjust to the new reality and new challenges. International students have to repeatedly readapt to the sociocultural context which adds additional stressors and anxiety. Therefore, this readjustment becomes a significant factor for students’ anxiety and is an even stronger predictor of anxiety compared to the COVID-19 experience in family and among friends, or the social support perceived by the family. In contrast, a successful sociocultural readjustment to the new environment accompanied by sufficient family support are crucial protective factors for international students dealing with different challenges and anxiety caused by transition and pandemics.

## Limitations

The size of the sample was not large because of the limited number of first-year and second-year international undergraduate students at this University. Also, this study was conducted only in one university and not all students participated in both measurements as not everyone was present onsite during the COVID-19 pandemic. In the majority of cases, it happened due to lockdown regulations and in some cases due to significant obstacles to leaving their home country and reaching Lithuania for studies. Participants of this study had very diverse ethnic backgrounds and nationalities which made it difficult to compare the results of this study with others where the sample represents one bigger national group. For some students, English was not their first language, which may have had an impact on the quality of their responses and how well they comprehended some questions. Also, in some countries, it is not socially acceptable to talk about and reveal mental health problems, which might have affected the truthfulness of some answers to this questionnaire. Future studies should consider such factors as discrimination, stress, acculturation strategies and others which might also play an important role in international students’ adjustment and psychological well-being.

## Conclusions

This study revealed the importance of sociocultural adjustment, social support perceived from families and COVID-19 experience for international medical science students’ anxiety level during the second year of studies. Interestingly, the sociocultural adjustment level which was reached after the first semester of the first year didn’t play any significant role later in the anxiety of the second-year international students. That suggests an idea that after such critical events as COVID-19 which disturbed the international students’ everyday life and a normal adjustment process, a new readjustment to the sociocultural environment is needed to help students to deal with different stressors and anxiety. Therefore, some university programs, such as mentorship and special adaptation courses should be implemented not only during the first year of studies but also later in the curriculum or extracurriculars of the international students. Also, to facilitate sociocultural adjustment in the host country, mixed groups of international and local students could be created. On-site studies and group work tasks including an explanation of the local and international context in such groups could increase multicultural contacts and communication which may lead to a more sufficient understanding of the new cultures and better sociocultural adjustment in general. To ensure a calmer and secure atmosphere for international students, it is worth conducting classes in smaller groups. In this case, it is easier to apply an individualized approach to each student, and also it is easier to notice if any student is having problems, and as a result, such students will be more often provided with appropriate educational or social-emotional assistance.

Furthermore, the current research indicates that social support perceived by families is inversely correlated with symptoms of anxiety, acting as a protective factor against international students’ anxiety during the COVID-19 pandemic.

Therefore, social support emerges as a vital resource for international students in navigating stressors during pandemic outbreaks.

Moreover, as the number of medical sciences students suffering from moderate or severe anxiety is quite high among international students and even students who had not been diagnosed with COVID-19 experienced notable levels of anxiety related to the pandemic, more attention to mental health services should be addressed to help students navigate through uncertainty and deal with mental health problems. The findings of this study could help universities and psychological support service providers and educators to better understand the process of students’ sociocultural adjustment during the first two years of their studies abroad and prepare for some critical situations such as pandemics and various other restrictions. This study could also be helpful for universities to develop effective international students support policies and strategies for the future.

## Data Availability

The datasets used and/or analysed during the current study are available from the corresponding author on reasonable request.
